# An integrated approach to flood risk management and spatial quality for a Netherlands’ river polder area

**DOI:** 10.1007/s11027-015-9675-7

**Published:** 2015-08-03

**Authors:** Anne Loes Nillesen, Matthijs Kok

**Affiliations:** 1 Delft University of Technology, Delft, The Netherlands; 2 Defacto Urbanism, Rotterdam, The Netherlands; 3 HKV Consultants, Lelystad, The Netherlands

**Keywords:** Flood risk management, Local individual risk, Integrated approach, Spatial quality, Room for the River approach, Dike-ring 16, Netherlands, Rhine-Meuse-Scheldt delta

## Introduction

Deltas throughout the world are confronted with increasing flood risks. Flood risk can be defined as the product of probability and consequences of flooding (Hall et al. 2003). Flood risk management strategies in effect aim to reduce the probability and/or consequences of flooding events. These strategies evolve as flood risks increase, driven by factors such as subsidence, climate change, population growth and economic development.

There is a strong relationship between flood risk management and spatial quality: New or improved flood defence infrastructure can have a significant impact on spatial quality, especially in urbanised deltas with (historic) built environments, such as the Netherlands (Klijn et al. 2013). Because of a growing appreciation of this relationship, spatial quality is increasingly incorporated in the objectives to be achieved in the development of flood risk management strategies.

Flood risk management strategies in the Netherlands traditionally focus on reducing the probability of flooding (Klijn, this issue). The country is divided in dike-ring areas, i.e. areas that are protected against flooding from rivers, major lakes and the North Sea, through closed systems of dikes, dunes, dams, barriers and natural high grounds.

Until recently, Dutch legislation defined protection standards for dike rings related to the exceedance probability of flood levels. These standards were originally established in the 1960s and based on risk analysis and cost-benefit analysis, including factors such as economic output of an area and opportunities for timely evacuation of inhabitants (Ten Brinke and Jonkman 2009). Applied homogeneously to entire dike rings, the protection standards varied between 1 in 250, i.e. designed for situations that occur once every 250 years, and 1 in 10,000 (Slomp 2012). The flood defences were evaluated periodically; if certain sections or components of a dike ring did not meet the standard, reinforcements were implemented. This approach is referred to in this paper as the uniform dike-ring approach.

In the uniform dike-ring approach, any negative effects on spatial quality as a consequence of reinforcements are managed locally by embedding these infrastructure works in the surrounding landscape and built environment. The flood defence strategy employed is leading and the role of spatial design is limited to fitting in the flood protection measure; as a consequence, spatial quality often remains adversely affected. The interventions that were required to meet the protection standards have been facing growing opposition because of the negative effects on spatial quality and a renewed appreciation of cultural and environmental values (Klijn et al. 2013).

Within this changing context, the Dutch Room for the River programme has been developed. After two major river flood events in the 1990s, the implementation programme started in 2006 and is planned to ensure that the main rivers in the Netherlands are able to safely discharge the 1:1250 per year design river floods of 16.000 m^3^ as of 2015. Compared to the uniform dike-ring approach, the Room for the River programme can be regarded a trend reversal from both a flood risk management perspective as well as the perspective of including spatial quality as second policy objective.

From a flood risk management perspective, the variety of alternative flood probability reduction measures has been increased by including measures that lower flood water levels by creating ‘room for the river’. This can either be achieved within the existing floodplain area by removing obstacles in the floodplain, or through deepening the riverbed or excavating the entire floodplain, or by enlarging the floodplain area by relocating embankments, creating bypasses or making detention areas (Alberts 2009). Although the primary objective of the room for the river approach is to comply with the same standards as applied in the uniform dike-ring approach, spatial quality is an important secondary objective (Demon and Alberts 2005). In order to address both objectives in the Room for the River Programme (room for the river), a more integrated approach to flood risk management and spatial quality was developed.

In the room for the river approach, first, an inventory of approximately 700 possible measures was made and assessed using multiple criteria that include spatial quality (Klijn et al. 2013). The assessment was facilitated through a qualitative assessment framework for spatial quality and supervised by a quality team. The outcomes of this assessment formed an integral part in decision-making processes. Ultimately, about 40 measures were selected that together accomplish sufficient reduction of the water levels of a certain river branch. While the room for the river programme specifically aimed to address the spatial quality both on the scale of entire river branches as well as the local scale of the intervention, the implementation by local governments shifted the focus of the integral design to the embedment on a local scale (Hulsker et al. 2011).

Although the inclusion of spatial quality in the room for the river approach is deemed successful, the number of flood risk protection options is limited to two: dike reinforcements and lowering the flood level by room for the river interventions, such as the aforementioned. Spatial quality and its associated economic costs only serve as evaluation criteria, provided that both options are hydraulically effective and thus valid alternatives.

**Fig. 1:**
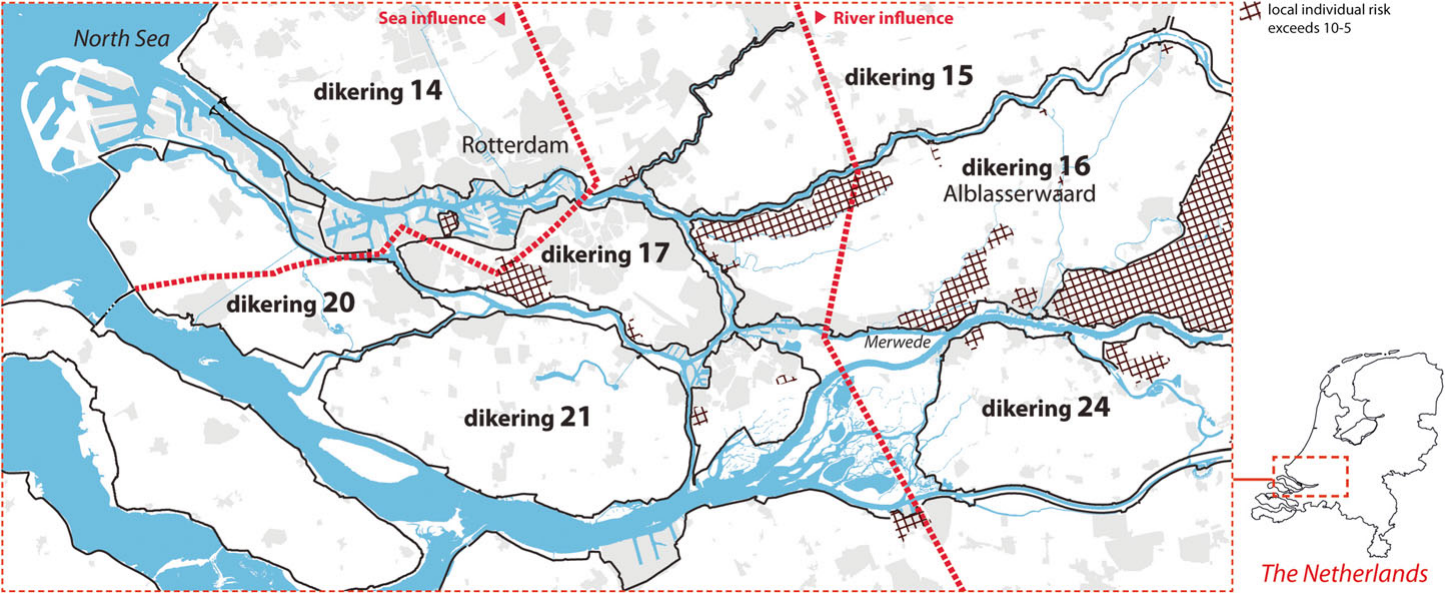
Dike rings in the Netherlands’ Rijnmond-Drechtsteden area. The hatches indicate the areas that currently do not comply with the standard

Recently, the Delta Programme was established in order to define future strategies for flood risk management in the Netherlands (Delta Commission 2008). In contrast to Room for the River, the programme addresses flood defence strategies not only for rivers but also for coastal areas and estuaries. The programme proposed a new risk-based standard, the basic safety level, also known as local individual risk (LIR). The new standard defines a maximum yearly probability for loss of life as a direct consequence of flooding, rather than a probability standard for flood defence structures. The new safety standard is set at 1/100.000 (10^−5^) and is to be complied with at any location within a dike-ring area (Deltaprogramma 2013). Areas that currently do not meet this standard are shown for the Netherlands’ Rijnmond-Drechtsteden region in Fig. 1 and require the development of new or additional flood defence strategies or measures.

As this paper will argue, the new risk-based standards create the opportunity for a truly integrated approach to flood risk management while enhancing spatial quality. Compared to the conventional protection standard for flood defence structures, the risk-based standard allows for a wider variety of possible flood risk measures. The availability of a variety of measures that can effectively address flood risk allows for spatial quality to become a decisive *ex-ante* criteria.

First, this paper will describe how the new risk-based flood risk standard allows for an integrated method for flood risk management and the enhancement of spatial quality. Underlying concepts such as differentiated dike rings and the basic safety level will be explained. Second, the proposed integral approach for developing flood defence strategies while enhancing spatial quality is described. Subsequently, the results of applying the integrated approach to the case study area of one of the Dutch dike-ring areas called the Alblasserwaard will be shown and compared with results from application of the uniform dike-ring and room for the river approaches.

## Materials and methods

### Principles underlying the integrated approach to flood risk management and spatial quality

The integrated approach proposed in this paper is based on two important principles: (1) An increase in the number of alternative, exchangeable flood risk reduction measures and (2) the *ex-ante* inclusion of a spatial quality assessment. First, it is explained how the introduction of the basic safety level extends the amount of applicable flood risk protection measures. Subsequently, the different steps of applying the integral approach, in which spatial quality is used as an *ex-ante* criterion, are described.

### Basic safety level and opportunities for a variety of exchangeable measures

Compared to the conventional flood probability standards, the new basic safety standard allows for a wider variety of possible and exchangeable flood risk reduction measures in two ways: (1) by widening the scope of potential measures by, in addition to probability reduction measures, including consequence reduction measures and by (2) by creating the possibility to define different standards per individual dike-ring segment, instead of applying one predefined standard to the complete dike ring.

The key principle behind this is that the basic safety level addresses risk, whereas both the uniform dike ring and Room for the River approaches address probability. Risk can be defined as probability × consequence; flood risk reduction can be achieved through measures that reduce the probability and/or consequences of flooding, and consequence reduction may thus substitute for probability reduction. Examples of consequence reduction measures are improved evacuation strategies, compartmentalization, ground elevation, adaptive building and emergency shelters.

In order to verify compliance with the basic safety level, a methodology has been adopted in the Delta Programme that takes into account both the probability and consequences of failure for each dike-ring segment (Stijnen et al. 2008). These consequences are calculated using flow pattern simulations during normative conditions, combined with the evacuation fraction of a dike ring (Deltares 2011). The flow pattern calculations indicate the flooding water levels in meters/h and maximum water depths during flooding; the evacuation fraction is an indication of the probability of people being present. Through this flow pattern analysis, the contribution of each individual dike-ring segment to overall flood risk can be estimated (Jongejan and Maaskant 2013). This allows for a differentiated dike-ring approach in which probability reduction measures can be evaluated and applied per individual dike segment.

Additionally, the flow pattern maps give insight in the direct geographical relation between the failure of a certain dike segment and the dispersion of the consequential flooding. This way, insight is provided into the required location of possible probability or consequence reduction measures, supporting the gathering of variety of possible measures.

### Integrated approach to flood risk and spatial quality

This paper proposes a new approach for addressing flood defence and spatial quality aspects in a coherent, integrated fashion. It is intended for use at the scale of a dike-ring area and is composed of four steps:

1. Selection of flood risk interventions that either have:
  - A positive effect on spatial quality and a considerable contribution to flood risk reduction, or
  - A neutral impact on spatial quality and a major contribution to flood risk reduction
2. Revision of the LIR assessment of the area, resulting in the definition of new or remaining focus points
3. Address the remaining LIR areas with a new round of flood risk interventions while using design optimization to embed the necessary interventions
4. Repeat steps 2 and 3 (until the LIR target is achieved)

The assessments on spatial quality and hydraulic effectiveness of different measures can both take place *ex-ante* and can provide criteria for inclusion of specific measures in a flood risk management strategy. Both the spatial assessment framework, which evaluates the positive, neutral or negative impact on spatial quality of an intervention, and the hydraulic effectiveness assessment, which evaluates the considerable or major contribution to flood risk protection, should always be adapted to the region of attention.

### Spatial quality assessment framework

A framework for assessing spatial quality was developed in the room for the river programme, necessitated by the inclusion of spatial quality as an evaluation criterion in that programme. The framework assumes that a so-called quality team is established that evaluates flood risk interventions for their impact on spatial quality, using a predefined set of criteria; given the background of the room for the river programme, the method is geared towards relatively rural river areas. It has been adjusted in previous research to be applicable in a somewhat more urban context, such as that of the Alblasserwaard and Rijnmond regions (Nillesen 2013). Derivatives of this framework are used for the evaluation of the different approaches in this paper.

### Methodology for evaluating the proposed new integrated approach

In this study, the uniform dike-ring approach, the room for the riverapproach and the proposed new integrated approach are applied to a case study area, the Alblasserwaard, also known as dike ring 16. Subsequently, it is evaluated if the latter method has a reduced negative impact on spatial quality in comparison to the first two methods.

Research steps:

- Application of the uniform dike-ring approach, based on the basic safety level, and reflection on spatial quality
- Application of the room for the river approach, based on the basic safety level, and reflection on spatial quality
- Application of the proposed new integrated approach, based on the basic safety level, and reflection on spatial quality
- Discussion of results

### Case study area: Alblasserwaard (dike ring 16)

Dike ring 16, the Alblasserwaard polder, serves as case study area. It is located east of the Rijnmond-Drechtsteden area, an urbanised and industrial region that includes the port of Rotterdam, the Netherlands (Meyer et al. 2012). The Diefdijk separates dike ring 16 from the adjacent dike ring 43, the Betuwe, and provides compartmentalization in case of a dike breach. The rivers Lek, Noord and Merwede form its other boundaries. Hydraulic boundary conditions are influenced by both North Sea water levels and peak river discharges towards the west; towards the east, peak river discharges are the dominant factor.

The land that is now Alblasserwaard was gradually reclaimed and embanked starting in the eleventh century. Eastern sections of the Alblasserwaard were part of the Hollandse Waterlinie; these are historic military defence works that date back to the seventeenth century and were comprised of fortresses and wide spreads of low lying land that could be inundated to prevent enemy intrusion. The polder has an extensive drainage system that is characterised by east to west canals that were originally drained at the north-western tip, by the windmills of Kinderdijk; nowadays a major tourist attraction, their function was gradually taken over by steam pumping stations and subsequently electric and diesel pumping stations. Originally used for agriculture, the land subsided so that at present, it is mostly used as grassland and livestock management (Steenbergen and Reh 2009).

**Fig. 2:**
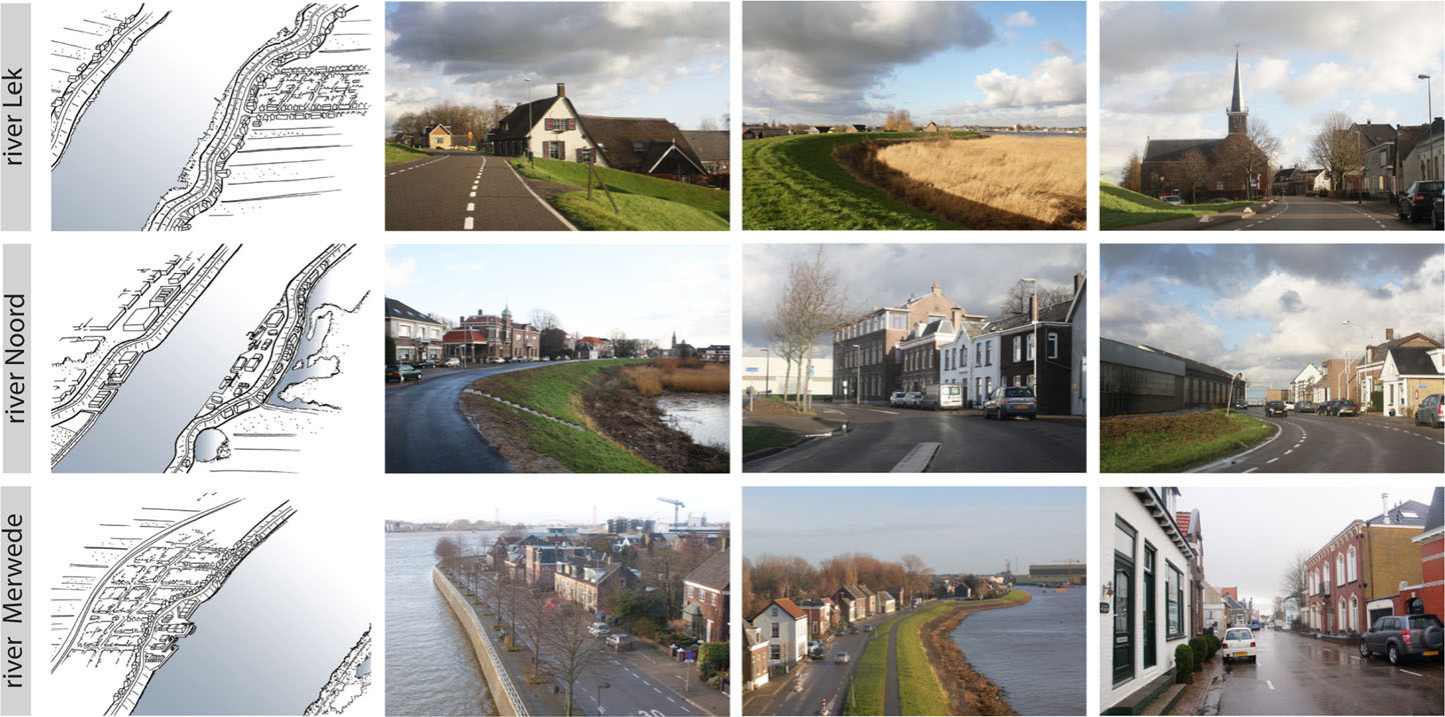
Images characterising the Ablasserwaard riverfronts

Different spatial characteristics can be found along each of the rivers. Figure 2 shows some characteristic images of the dikes around the Alblasserwaard. Along the river Merwede, in the south of the polder, the dikes are densely built; historic dike ribbons can be found here, with buildings on both sides of the dike. This is the economic and urban centre of the Alblasserwaard, with dredging and transhipment companies and shipyards in the unembanked areas. A clear view over the river is a rare but appreciated condition. Along the river Noord, in the west, historic ribbon development limits the view to the polder itself. Unembanked areas along the riverside of the dikes contain a mix of industrial buildings and natural reed beds. The urban and industrial areas at the confluence of Merwede and Noord rivers are part of the Drechtsteden cluster of ports. Along the Lek, in the North, individual houses and villages can be found. The riverside of the dikes along the Lek is predominantly unbuilt and consists of reed beds. The infrastructure route on the dike provides wide views to both polder and river.

The polder is characterised by built dike ribbons along the rivers that surround open polder landscapes (Steenbergen and Reh 2009), with small towns and an economy based on agriculture, livestock and river bound activities. A restrictive building policy is applicable that aims to preserve the open character of the landscape. Dike ribbons can be found not only along the rivers but also within the polder, along some of the historic peat and drainage canals. Two important infrastructure corridors cut through the Alblasserwaard: from west to east, the A15 national highway and the international Betuwe freight train line and from north to south the Netherlands national A27 highway. Along those national highways, clustered commercial developments occurred. The polder has a population of approximately 170.000 inhabitants, with a density of approximately 680 inhabitants/km^2^.

### Application of the uniform dike-ring approach

In this approach, the flood risk reduction that is required to meet the basic safety level is achieved through increasing the failure probability standard for the Alblasserwaard dike ring. Expert judgement was involved to determine this standard. Compliance of dike-ring sections and components with the adjusted failure probability standard is evaluated; any infrastructure upgrades or reinforcements that are required to meet the adjusted standard are determined with the help of expert judgement. The impact of these infrastructure works on spatial quality is subsequently assessed.

Given the time-consuming and costly nature of fully invoking the spatial quality assessment method from the Room for the River programme, in this study a similar but less elaborate expert workshop was used to determine and evaluate the effects of the necessary dike reinforcements from the uniform dike-ring approach on spatial quality. Eighteen subject matter experts, local representatives and officials assessed the impact of potential dike reinforcements on spatial quality. The participants were assigned to one of three groups, each focussing on one of the rivers and corresponding dikes that run along the Alblasserwaard. Large printouts were available explaining the context for the Lek, Noord and Merwede:

- A large aerial picture of each river on a 1:10.000 scale
- Photos and street view images, showing the local situation at approx. every 1000 m along the dikes
- An inventory of the proximity of buildings to the dike body. The inventory displayed whether there are buildings situated on top of the dike, in the inner dike slope, the outer dike slope or at the foot of the dike

A hydraulic engineering/technology expert was included in each group. One group leader guided the discussion and marked the groups’ assessment, indicated the threshold height for the indicated spatial assessment, and main arguments. The spatial assessment was provided and collected in a relatively simple qualitative fashion:

- “+” for a positive effect on spatial quality (the proposed intervention offers an opportunity for the improvement of spatial quality or offers opportunities for synergy)
- “0” for a neutral effect on spatial quality (the proposed intervention does not have a positive nor a negative impact on spatial quality)
- “-“ for a negative effect on spatial quality (the new situation including the proposed intervention will be less qualitative as compared to the current situation, e.g. the potential intervention blocks the line of sight to the river.)
- “--“ for a very negative effect on spatial quality (e.g. the interventions require characteristic housing along the dike (ribbon development) to be demolished.)

### Application of the Room for the River approach

The method for evaluating the impact of the room for the river approach builds forth on the results from the uniform dike-ring approach. In addition to measures developed in that approach, alternative Room for the River interventions are identified for the sections of the dike ring where normative water levels are dominated by peak river discharges. This concerns the south-eastern part of the Alblasserwaard dike-ring area.

The hydraulic effectiveness of these measures was determined using expert judgement. The effectiveness of potential load reducing measures was assessed in an expert workshop that consisted of two separate sessions:

- One session focussed on producing an inventory of potential hydraulic load reducing interventions for the Alblasserwaard dike-ring area. As a preparation, measures from previous room for the river programme studies were identified through desk study. During the workshop, these were complemented by expert judgement.
- One session, with three hydraulic engineers, focussed on the assessment of the hydraulic effectiveness of the potential load reducing measures. The measures identified in this session as being effective were subsequently verified with additional hydraulic calculations (Van Putten 2013).

A total number of 23 measures was identified and hydraulically assessed. In this paper, only those measures are indicated that are effective alternatives for dike elevation. The entire inventory and assessment can be found in a workshop report (Defacto 2013).

The spatial assessment regarding the potential load reducing measures was organised in a separate session with 20 participants, including subject matter experts and local representatives. Participants quantified the expected impact on spatial quality of an intervention as positive, neutral, negative or very negative and explained their reasoning.

### Application of the integrated approach

The first step in the application of this approach is the selection of flood risk interventions that either:

- Have a positive effect on spatial quality and a considerable contribution to flood risk reduction, or
- Have a neutral impact on spatial quality and a major contribution to flood risk reduction

In order to do so, possible measures for probability or consequence reduction have to be identified, and an *ex-ante* analysis has to be carried out on the effectiveness from the perspectives of flood risk management and spatial quality.

#### Effect of interventions on flood risk reduction

In order to determine which interventions effectively reduce flood risk and to what extent, it is important that the interventions address potential failure mechanisms that contribute to flood risk: specifically, to LIR and the number of fatalities. This allows for considering the effects of both probability and consequence reduction measures.

Step 1 of the integrated approach involves determining the effects of possible interventions on flood risk and spatial quality. Data on the effectiveness of probability reduction measures was available, but data on consequence reduction measures in relation to flood risk reduction and spatial quality had to be obtained. The first round of the study therefore focussed on the effects of reinforcing multiple dike sections and potential load reducing interventions. Compared to the other two approaches, the number of possible interventions was increased by taking into account the opportunity to reinforce dike-ring segments in a differentiated fashion, instead of applying a homogenous standard, and through assessing their contribution to the consequences of a flood.

**Fig. 3:**
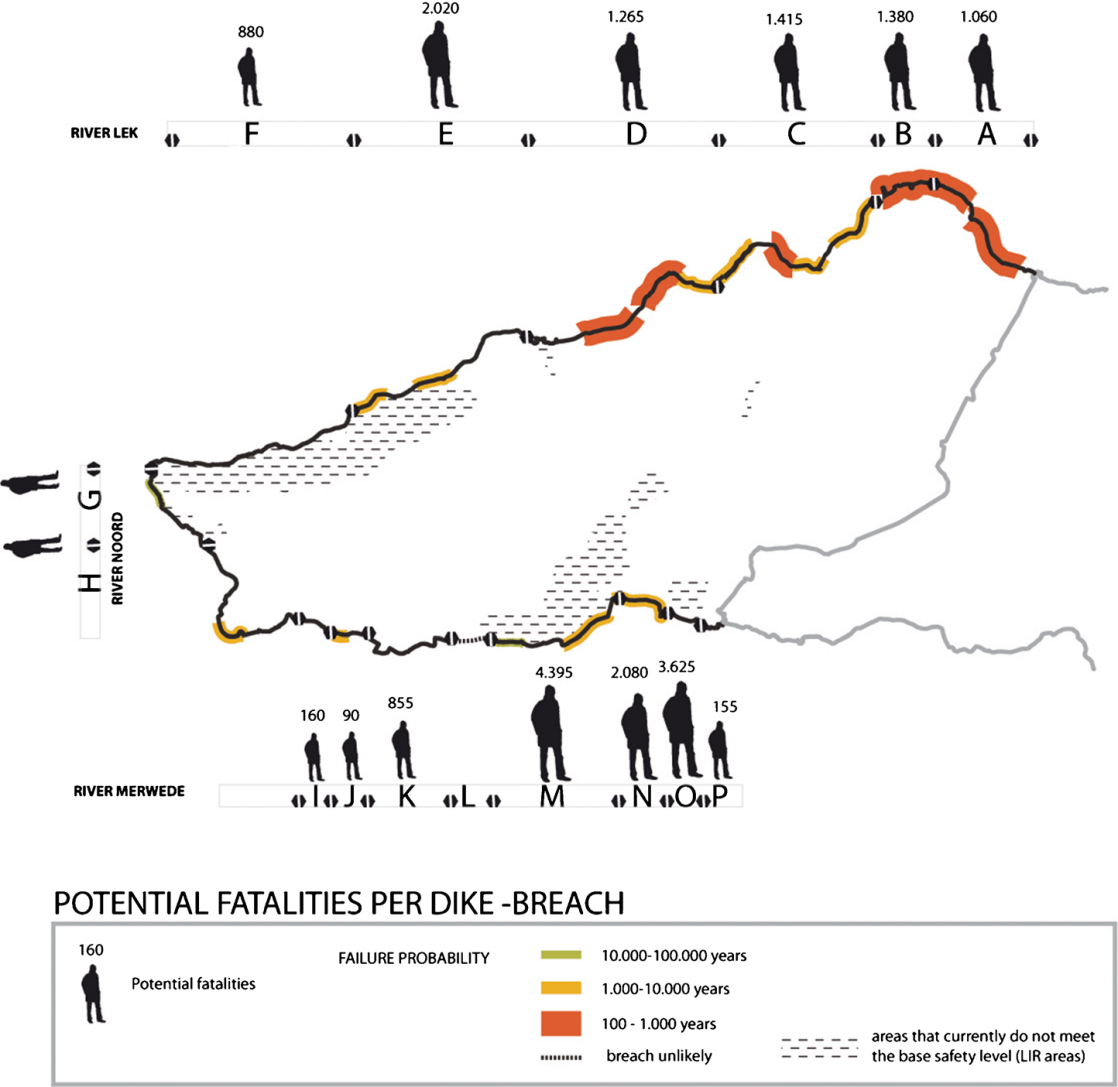
Failure probabilities as indicated by VNK (Vergouwe & Berg 2013), and an overview of the number of fatalities after dike failure

**Fig. 4:**
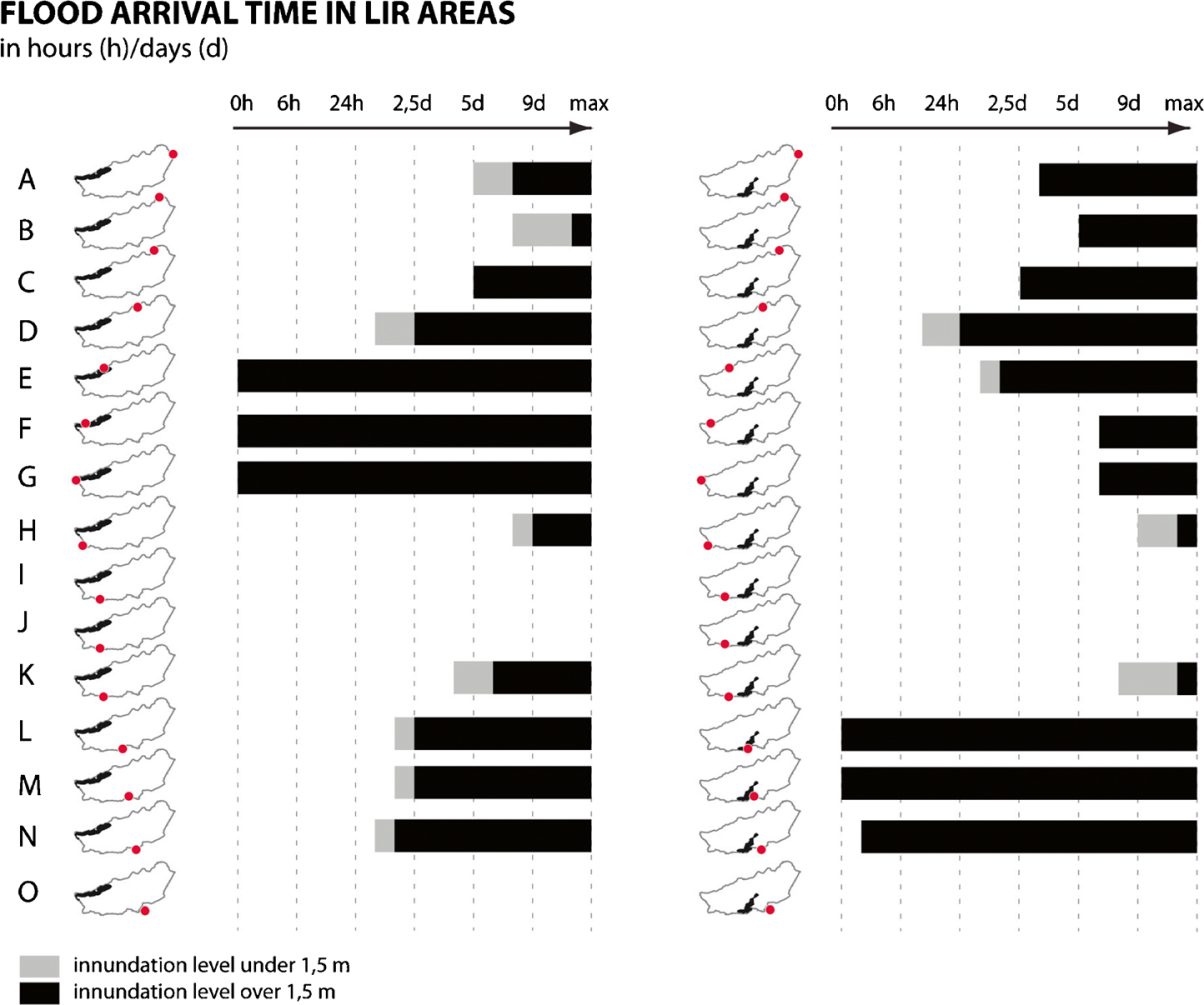
Overview of the time it takes for a flood to reach the main LIR areas

For the probability reduction measures related to dike reinforcements, first, the current protection levels of the dike segments are indicated by their individual failure probabilities; to this end, the preliminary results of the VNK study (Vergouwe and Van den Berg 2013) are employed (Fig. 3) To assess the share that a dike breach in a single dike section has in the total consequential damage for the entire dike-ring area, we focus on the consequential damage of a flooding event as expressed in LIR and potential fatalities, the following data is employed:

- An overview of the fatalities expected after dike failure, presented for all of the Alblasserwaard dike sections, based on hydraulic simulations of flow patterns (Fig. 3).
- An overview of the time it takes for a flood to reach the two different LIR areas (in grey) after a dike breach (listed in the remainder of the paper as arrival time). And, in black, the time in which the inundation level in the LIR area reaches 1.5 m (Fig. 4).

The dike segments are categorised according to which potential flood risk interventions are indicated to either have a major or a relevant effect on the reduction of dike-ring area’s flood risk^2^.

Major effect: interventions that reinforce or are load-reducing, dike segments that, in case of a breach, would be characterised by:

- A very short arrival time; inundation levels in one or more LIR areas are over 1.5 m within a 6-h time span, combined with a moderate dike section failure probability of over 1/10.000
- Considerable number of fatalities (>1000 persons), combined with a very high dike section failure probability (over 1/1.000)

Relevant effect: interventions that reinforce or are load reducing, dike segments that, in case of a breach, would be characterised by:

- A short arrival time (<24 h); inundation levels in one or more LIR areas are over 1.5 m within a 24-h time span, combined with a moderate dike section failure probability of over 1/10.000
- A considerable number of fatalities (>1000 persons), combined with a considerable dike section failure probability (1/1.000 to 1/10.000)
- A high number of fatalities (>5000 persons), combined with a high dike section failure probability (1/100 to 1/1.000)

Step 2: Update the LIR assessment, involves expert judgement by a civil engineer to estimate the remaining flood risk assignment and results in the definition of new or remaining focus points.

Step 3 of the method involves consideration of additional flood consequence reduction measures for the second round of selecting flood risk interventions, in order to address the potential LIR areas where the basic safety level is not met. This is a creative aspect of the process, supported by indicative expert judgements, that illustrates how a wider range of interventions is included in the development of an integral flood risk strategy.

### Effect of interventions on spatial quality

The performed ex-post spatial impact assessments of the dike reinforcements in the applied uniform dike-ring approach and the load reduction measures in the applied Room for the River approach were used to represent the *ex-ante* spatial quality assessment for the integrated approach. This helps identify which possible dike segment reinforcements have a positive, neutral or negative impact on spatial quality.

## Results and discussion

Results of the uniform dike-ring approach, Room for the River approach and integral approach are explained below.

### Meeting the basic safety standard with the uniform dike-ring approach

**Fig. 5:**
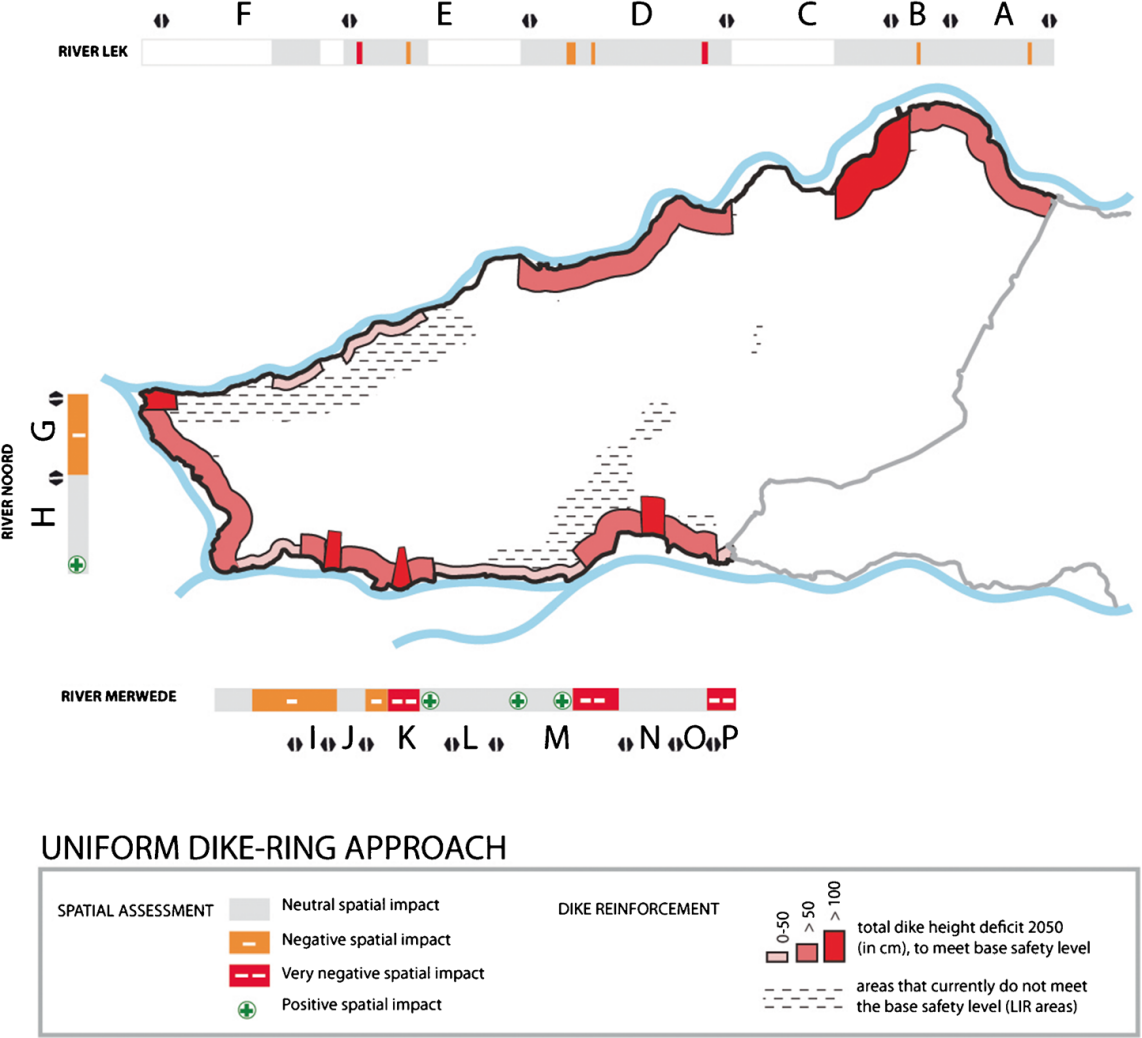
Dike elevations necessary to meet the LIR standard by following the uniform dike ring approach

Areas within the Rijnmond-Drechtsteden region that currently fail to meet the new basic safety level (LIR areas) are shown in Fig. 1. According to these estimates, the safety standard would have to be increased with 26 times for the Alblasserwaard dike-ring area, in order to meet the required basic safety level (Slootjes and Jeuken 2013, p. B09). Figure 5 visualises the required dike elevations in centimetres that would be necessary to meet the new standards by 2050 for the Alblasserwaard, based on expert judgement.

As earlier dike elevations and reinforcements to meet the previous probability-based standards were problematic, additional elevations necessary to meet the basic safety standard will not only be costly—estimates indicate € 1.520.000.000—(Slootjes and Jeuken 2013, p. B11) but will also have a major impact on spatial quality in the region. The outcome of the spatial assessment workshops regarding the impact of potential dike enforcements on spatial quality is also shown in Fig. 5. Negative effects can be found along the characteristic and historic dike ribbons along the Merwede and Noord rivers. Along the Lek, buildings with high historic-cultural value are affected. Along the Merwede river, some positive effects and possibilities for synergy were found.

### Meeting the basic safety standard with the Room for the River approach

A total of 23 (combinations of) measures was brought forward in the exploratory workshop for identifying potential load reducing measures. These measures were assessed for their hydraulic effectiveness; 19 measures were expected to have a positive hydraulic effect. The load reducing measures are concentrated along the Merwede river in the southeast of the Alblasserwaard, in the area where normative water levels are dominated by peak river discharges.

**Fig. 6:**
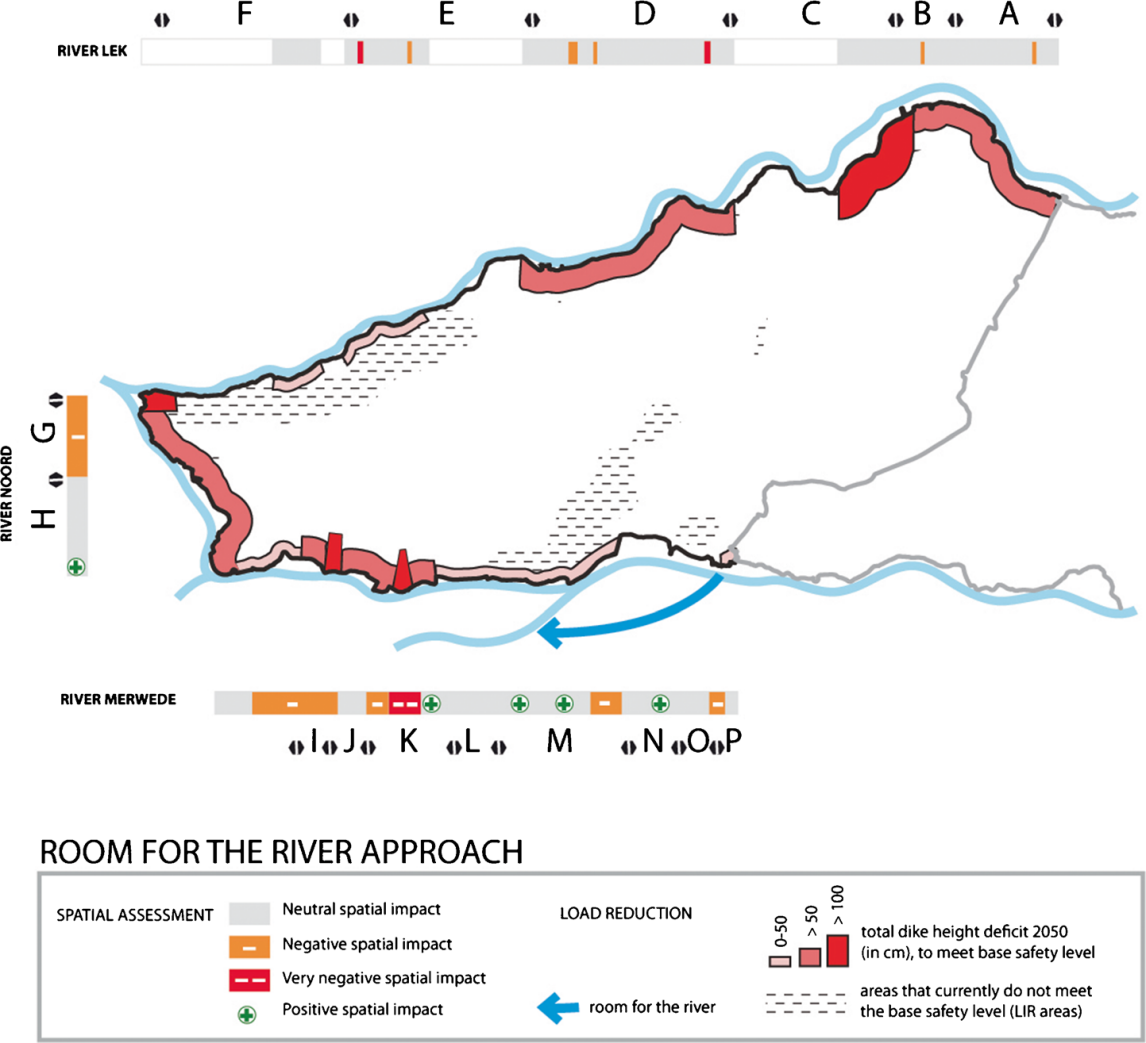
Probability reduction measures necessary to achieve the LIR standard by following the Room for the River approach

Only a few measures were found to be effective alternatives for dike reinforcement. During the qualitative assessment workshop, one of these measures (illustrated in Fig. 6) was presented to have a potential positive effect on the areas’ spatial quality. This concerns the creation of a bypass through the polder south of the Alblasserwaard, in parallel to the Merwede, and can be combined with the development of a new harbour for the village of Werkendam. This measure could serve as an alternative for two dike reinforcements in dike ring 16 that have a very negative impact on spatial quality.

### Meeting the basic safety standard with the integrated approach

Two areas in the Alblasserwaard polder do not meet the basic safety level of 10^−5^: an area in the north-western part of the polder along the Lek river, and a centrally located area in the south, along the Merwede river. Figure 4 illustrates how a dike breach (dike failure) in each of the corresponding dike sections would contribute to flood risk in these LIR areas; these areas are under threat of flooding regardless of the location of a breach.

- Six dike segments contribute to flooding instantly to an inundation level of up to 1.5 m. These segments are therefore major contributors to the emergence of LIR areas. For the north-west LIR area, these segments include section E, F and G. For the southern LIR area, these are the dike segments L, M and N. These dike segments are thus said to have a very short arrival time.
- Flood waters entering the polder through a breach in dike segment D would reach the southern LIR area within approximately 16 h and lead to an inundation level of 1, 5 m within approximately 24 h. This dike segment is therefore indicated to have a short arrival time.

Many dike segments have low failure probabilities. Dike segments A and B and parts of dike segments C and D have a failure probability between 1/100 and 1/1000. Parts of the dike segments of C, D, E, H, J, M and N have a failure probability between 1/1000 and 1/10,000. So-called piping is the main failure mechanism of the dike segments.

**Fig. 7:**
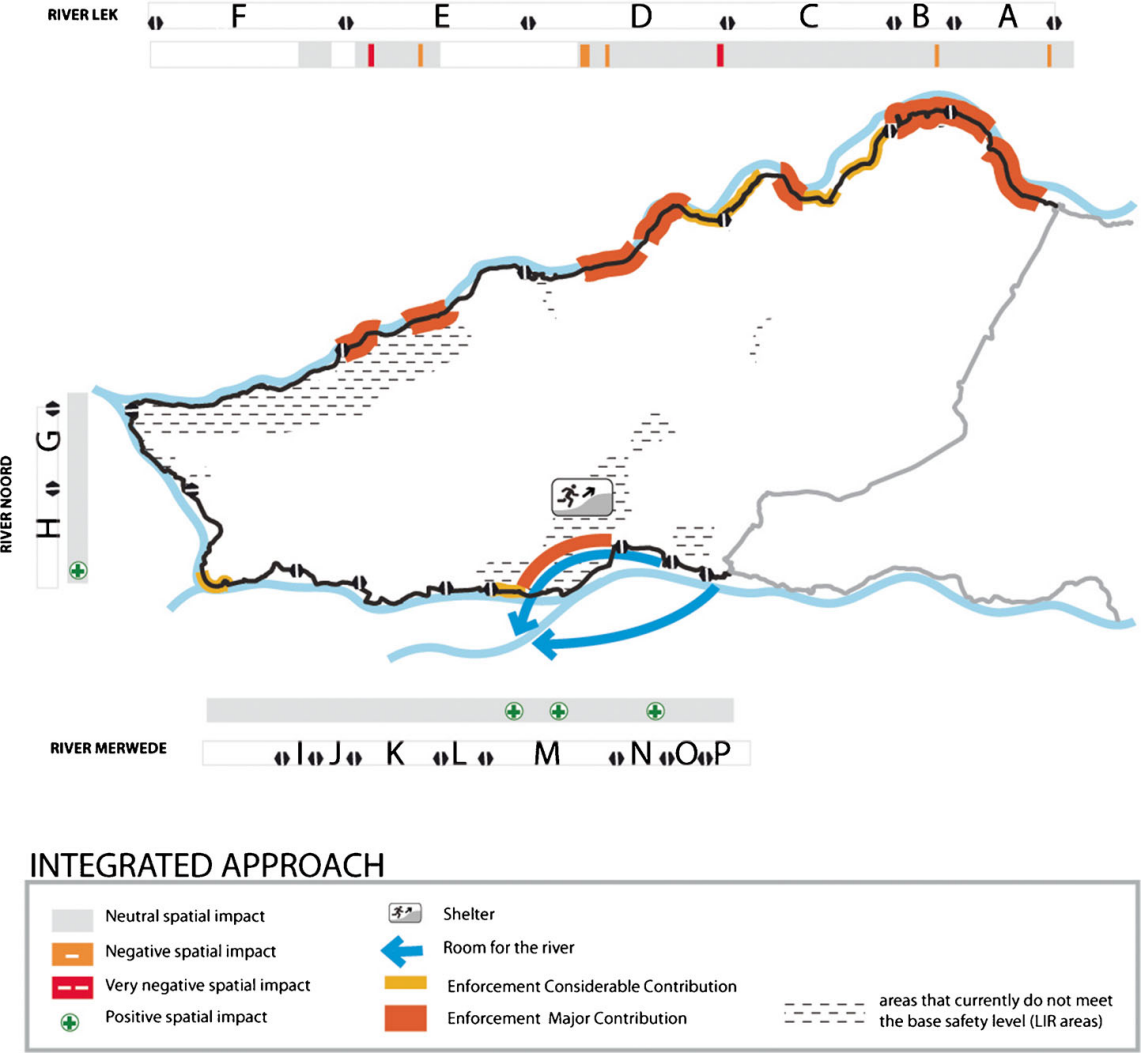
Probability and consequence reduction measures applied to achieve the LIR standard by use of the integral approach

When combining failure probabilities with potential consequences of flooding, dike reinforcements or load reducing measures is a priority for dike segments indicated in Fig. 7.

For the spatial assessment, the outcome of the ex-post assessment of the dike reinforcements from the uniform dike-ring approach is used as *ex-ante* impact criteria of potential dike elevations. In addition, the 19 effective load reduction measures from the room for the river approach were evaluated in a similar fashion for their impact on spatial quality as an *ex-ante* step. One measure is selected in addition to the measure selected earlier in the room for the river application; the river bypass combined with a harbour in the dike-ring area south of the Alblasserwaard (dike ring 24). The second spatial potential that was addressed is to combine the construction of a new river bypass along an existing canal with the necessary broadening of the highway along the canal inland of dike segment M.

### Step 1: Selection of measures from a flood risk and spatial quality perspective

The combination of a Room for the River intervention with the construction of an additional harbour in dike ring 24 is considered to have a positive effect on spatial quality as well as a considerable effect on flood risk reduction in the Alblasserwaard. The load reducing intervention is expected to lower the water level of the Merwede during normative conditions with approximately 40 cm near dike segment P.

Along the Lek, most dike reinforcements that have a major impact on risk reduction have a neutral impact on spatial quality, except for some locations in which individual buildings are in close proximity to the dike. In case an individual building needs to be spared from demolition, it is possible to choose for a dike enforcement construction, using sheet piling, reducing the footprint needed for the dike reconstruction. The dike segments with a major contribution to flood risk reduction along sections A, B and C have enough space available to bring the levee up to its standard, increase the standard by enforcing it 26 times and even to construct a dike with a 1/100,000 failure probability, while maintaining an acceptable level of spatial quality.

### Step 2: Revision of the LIR map

Expert judgement indicated that the interventions as described up to this point will result in a significant reduction of flood risk in the Albasserwaard. As a result, the LIR area in the north, along the Lek river, now complies to the required basic safety level. However, the dike segment M remains as an essential dike segment to be reinforced in order to fully address the LIR area to the south. Dike reinforcement along dike segment M is indicated to have a very negative impact on spatial quality. The main argument is that many characteristic buildings in close proximity to the dike body are found along the dike’s polder side. Dike reinforcement at this location cannot be realised towards the river side of the dike either since the dike already contributes to the existence of a critical bottleneck in the Merwede river.

### Design optimization

Further design optimization is needed to handle the dike section M and the related southern LIR area. Two alternatives are put forward. The first alternative consists of the construction of a cofferdam that strongly limits the space needed for dike reinforcement. A cofferdam is a large construction and can be designed to match a failure probability of 1/100.000. The rough cost indication of this type of dike is 18.700 euros a meter (Deltares, 2013), excluding the potential costs for the demolition of houses along the dike. This would result in a cost estimation of 43 million euros for the reinforcement of the 2.3 km long dike segment.

An alternative is to combine the Room for the River measure of creating a bypass out of the nearby Steenenhoek channel with dike relocation. The Room for the River measure decreases water levels around dike segment P with approximately 14 cm. With that, dike ring 16 is slightly reduced in size and the new dike, with a 1/100.000 failure probability, can be combined with the broadening of the highway further inland. This creates a new, small dike-ring area. The cost of this intervention is estimated at approximately 38 million Euros. This dike-ring area will have a high LIR since its small surface is quickly inundated when flooding. The small dike-ring area is positioned in the river dominated area, in which high water levels can be reliably predicted some days in advance. This makes it is possible to evacuate this new dike-ring area timely. In general, the evacuation fraction for dike-ring areas in the river dominated area is 75 %, instead of the 15 % that is now used for the partly sea dominated flood risk rivers along of the Alblasserwaard dike-ring area. A higher evacuation fraction reduces the consequences of a flood and with that the LIR.

As assessed by expert judgement, increasing the failure probability to 1/100.000 along the dike segment M, the southern LIR area will be resolved. For the design variant including the dike relocation combined with a room for the river measure, it is not yet determined whether it will address the LIR area sufficiently. If necessary, consequence reduction measures, such as flood shelters, can be applied in the potentially remaining LIR areas to further remain the LIR.

## Conclusions and recommendations

This paper introduced an integrated approach to flood risk management in which spatial quality is included *ex-ante*. Compared to the uniform dike-ring and room for the river approaches, two alternatives used in the Netherlands, this new approach offers a flood risk management strategy that reduces the negative impact on spatial quality of the interventions that are necessary to meet the basic safety standard.

In generic terms, applying a risk-based approach allows the inclusion of both probability reduction as well as consequence reduction measures in flood risk reduction strategies. This has two main advantages:

Firstly, in comparison with a one-sided probability reduction-based approach, as applied for instance in the Netherlands, Indonesia and Thailand, the number of potentially suitable flood risk reduction measures is increased. This broader availability of effective flood risk reduction measures allows for spatial quality to be applied as an *ex-ante* criterion for selecting measures. The inclusion of the impact on spatial quality of interventions as an *ex-ante* criteria in the formation of the flood risk strategy allows spatial planners to be involved in an earlier research stage in which the flood risk management strategy is formed, instead of solely embedding a given regional flood risk strategy ex-post.

Secondly, the method offers a valuable framework for developing a combined probability and consequence reduction strategy. This allows the method to become a valuable decision-making tool in so-called multi-layered flood risk management approaches, in which interventions regarding the probability and the consequential damage of a flood are combined (Hoss et al. 2011). Multi-layered safety approaches have often been referred to in the flood risk management debate, but so far, consistent methods for achieving a balanced probability and consequence reduction strategy were not in place.

In order to utilise the proposed methodology, quantitative data and/or qualitative expert guesses on both the effectiveness of probability reduction as well as consequence reduction measures should be available. Given the Dutch setting of this study, a country with a strong focus on probability reduction and a lack of data on the effectiveness of consequence reduction measures, the consequence reduction measures were subordinate to the probability reduction.

The method would be valuable to apply in Deltas with a risk-based flood risk protection target, where the formulation for a flood risk management strategy is ongoing and both probability as well as consequence reduction measures can be considered in a multi-layered flood risk management approach. In order to be applied in another geographical and cultural context, the assessment of the effectiveness of flood risk reduction measures and the assessment of the impact on spatial quality of measures should be adjusted and calibrated to fit the local values.

### Notes

Similar systems can be found in the United States, Indonesia and Thailand; the physical characteristics of such protection-centred approaches are almost identical (CIRIA, 2013).

2 A scenario in which multiple simultaneous dike segment failures occur is not discussed in this study.
